# Activity and safety of KEES - an oral multi-drug chemo-hormonal metronomic combination regimen in metastatic castration-resistant prostate cancer

**DOI:** 10.1186/s12885-023-10780-y

**Published:** 2023-04-04

**Authors:** Mustafa Asowed, Nils O Elander, Linn Pettersson, Maria Ekholm, Dimitrios Papantoniou

**Affiliations:** 1 Department of Biomedical and Clinical Sciences, Linköping University, Linköping, 581 83 Sweden; 2 Department of Oncology, Ryhov County Hospital, Jönköping, 551 85 Sweden; 3 Department of Medical Sciences, Endocrine Oncology, Uppsala University, Uppsala, 751 85 Sweden

**Keywords:** Prostate cancer, Metronomic chemotherapy, Cyclophosphamide, Ketoconazole, Estramustine, Etoposide

## Abstract

**Background:**

Metastatic castration-resistant prostate cancer (mCRPC) remains a therapeutic challenge and evidence for late-line treatments in real-life is limited. The present study investigates the efficacy and safety of an oral metronomic chemo-hormonal regimen including cyclophosphamide, etoposide, estramustine, ketoconazole and prednisolone (KEES) administered in a consecutive biweekly schedule.

**Methods:**

A retrospective cohort study in two Swedish regions was conducted. Overall (OS) and progression-free survival (PFS), biochemical response rate (bRR) and toxicities were analyzed.

**Results:**

One hundred and twenty-three patients treated with KEES after initial treatment with at least a taxane or an androgen-receptor targeting agents (ARTA) were identified. Of those, 95 (77%) had received both agents and were the primary analysis population. Median (95% CI) OS and PFS in the pre-treated population were 12.3 (10.1–15.0) and 4.4 (3.8–5.5) months, respectively. Biochemical response, defined as ≥ 50% prostate-specific antigen (PSA) reduction, occurred in 26 patients (29%), and any PSA reduction in 59 (65%). PFS was independent of prior treatments used, and KEES seemed to be effective in late treatment lines. The bRR was higher compared to historical data of metronomic treatments in docetaxel and ARTA pre-treated populations. In multivariable analyses, performance status (PS) ≥ 2 and increasing alkaline phosphatase (ALP) predicted for worse OS. Nausea, fatigue, thromboembolic events and bone marrow suppression were the predominant toxicities.

**Conclusions:**

KEES demonstrated meaningful efficacy in heavily pre-treated CRPC patients, especially those with PS 0–1 and lower baseline ALP, and had an acceptable toxicity profile.

## Introduction

Prostate cancer is the second most commonly diagnosed cancer, and the fifth most common cause of cancer-related death among men [1]. Established treatment options with proven survival benefit for metastatic castration-resistant prostate cancer (mCRPC) include taxanes (docetaxel, cabazitaxel), newer androgen receptor targeted agents (ARTA: abiraterone and enzalutamide) and radium-223 [2–9]. Recently, a poly(adenosine diphosphate-ribose) polymerase (PARP) inhibitor (olaparib), a new radioactive drug (Lutetium-PSMA) and several new ARTAs demonstrated encouraging outcomes in clinical trials [10–14]. In addition, a substantial amount of research has shown that more intense treatment approaches, rather than castration only, during the early castration sensitive phase improve survival [15]. Nonetheless, real-world studies have shown that overall survival (OS) in the mCRPC setting remains limited to 31 months in treatment-naïve patients, and only one year in patients progressing on docetaxel [16]. It is not uncommon for patients to run out of established treatment options, while still being in adequate performance status. Not surprisingly, unconventional and less documented treatment regimens have therefore frequently been used in this setting.

Low cost, acceptable toxicity profile, and oral administration make metronomic chemotherapy or chemo-hormonal therapy an attractive option in these cases. The concept of metronomic chemotherapy refers to a low-dose of frequently or continuously administered treatment with no extended interruptions [17]. In contrast to conventional chemotherapy regimens, usually administered at the highest tolerated doses, metronomic chemotherapy is characterized by lower, continuous plasma concentrations of the active agents. At these doses, certain chemotherapeutics may exert their anti-tumoral activity through stroma interactions and inhibition of angiogenesis, while retaining a favorable toxicity profile [18, 19].

Several small, retrospective studies show a modest effect of metronomic treatment in mCRPC. Single oral cyclophosphamide in combination with low dose corticosteroids is the most studied regimen [20–30]. Other combinations include vinorelbine, celecoxib, tegafur-uracil, methotrexate and lenalidomide [31–39].

A metronomic combination of oral cyclophosphamide, etoposide, ketoconazole, estramustine and prednisolone (KEES) was suggested to be an effective and tolerable regimen in a single arm, prospective trial with 17 patients by Jellvert et al. [40]. The choice of drugs used in the regimen was based on available agents at the time of the study, which had previously shown anti-tumoral activity in prostate cancer. The authors modified an older chemo-hormonal combination of ketoconazole, doxorubicin, vinblastine and estramustine [41] with a purpose of creating an orally administered, low-toxicity regimen, which could be used on an entirely outpatient basis [40]. Several centers in Sweden, including the oncology departments in Linköping and Jönköping, have offered KEES as a ‘last resort’ option in patients who have progressed on all standard therapies or have been ineligible for other standard treatments, and who remain in good performance status. The primary aim of this study was to investigate treatment efficacy, toxicity and prognostic factors in a large real-world cohort of mCRPC patients treated with KEES. To the best of our knowledge, this is the so far largest and most detailed published report evaluating any metronomic treatment in prostate cancer.

## Materials and methods

### Patients and data

In this retrospective cohort study, 123 consecutive patients with mCRPC who had previously received at least one line of taxane or ARTA and were treated with KEES at the Department of Oncology, Linköping University Hospital and at the Department of Oncology, Ryhov County Hospital from 2011 to 2020 were eligible for inclusion. Data on patients’ clinical status, treatments given, imaging and laboratory tests and on treatment toxicity were extracted from the hospitals’ medical records. Survival status was censored on September 30, 2022 or at last known contact. Gleason score was reported as per the International Society of Urological Pathology (ISUP) grade 1–5. Toxicities were retrospectively graded according to common terminology criteria for adverse events (CTCAE) v.4.0 and the highest grade reported was registered.

### Treatment

KEES was administered on a two-weekly cycle. During week one, cyclophosphamide 50 mg was administered two times a day and ketoconazole 200 mg three times a day. During week two, estramustine 140 mg and etoposide 50 mg were administered twice daily. Prednisolone 10 mg once daily was given continuously. All drugs were orally administered. At the physician’s discretion, treatment was initiated either continuously, as a six week schedule followed by two weeks’ pause (as was the case in the original publication by Jellvert et al. [27]), or as a two week schedule followed by one week’s pause. Dose reduction and/or discontinuation of one or more of the treatment components was possible and treatment beyond progression was allowed, if considered clinically beneficial by the treating physician.

### Statistical methods

Statistical analyses were performed with R version 4.1.2 (R Foundation for Statistical Computing, Vienna, Austria), using standard methodology (chi-square test for dichotomous variables, t-test or Kruskal-Wallis test for continuous variables and semi-parametric Cox models for censored variables). The sample size was not based on power calculations. Descriptive statistics were used. OS and progression-free survival (PFS) were analyzed for each treatment given using the Kaplan-Meier method, and between-group differences were compared using the log-rank test. Hazard ratios (HR) and confidence intervals (CI) for censored variables were estimated from Cox proportional hazards models. The proportional hazards assumption was tested. All tests were two-sided. P values < 0.05 were considered statistically significant.

#### Endpoints

Primary analysis population was defined as the patients having been treated with at least one taxane and one ARTA (“modern” population). Primary endpoint was OS in the primary analysis population. Secondary endpoints included biochemical response rate (bRR) and progression-free survival (PFS) in the same population, and OS, bRR, PFS and toxicities in the overall population. OS and PFS were defined as time from baseline to death from any cause or progression, respectively. A progression event was defined as either radiological progression, a rise of PSA of > 25% from baseline or nadir, unequivocal clinical deterioration, or death from any cause. In certain cases of long treatment responses with planned treatment pauses, PSA tended to rise shortly after the treatment was paused and decline at treatment restart. In these cases the date of definite PSA progression was used. Biochemical response was defined as PSA reduction of at least 50% from baseline.

#### Regression analysis

Univariable and multivariable cox regression were performed to examine the association between baseline characteristics and treatment effect in the overall population. As most patients were deceased at the time for data cut-off, we assumed at least 100 events in the Cox model. The following seven clinical prognostic factors were chosen, based on published literature [16, 42, 43], for evaluation in both the univariable and multivariable model: Age at treatment start, prostate-specific antigen (PSA), alkaline phosphatase (ALP), time from androgen deprivation therapy (ADT) to castration resistance, Eastern cooperative oncology group performance status (ECOG PS), visceral metastases and ISUP score. Line of treatment was considered, grouped as 2nd /3rd versus ≥ 4th line. Other clinical prognostic factors, including baseline hemoglobin, lactate dehydrogenase and pain [16, 30, 42, 43] were not pre-specified for data collection and not tested in the model. Treatment period (before vs. after 2015) was used as a stratification factor, as at approximately this time the adoption of cabazitaxel increased.

### Ethics

This study was approved by the Linköping ethical review board (Dnr 2018/139 − 31) and was conducted according to the Helsinki declaration. Due to the retrospective non-interventional design and the fact that the vast majority of patients were not expected to be alive at the time of data collection, the Ethics board waived the requirement for informed consent.

## Results

### Demographics

One hundred and twenty-three patients with prostate adenocarcinoma were included in the present study. Seventy-one patients (59%) had high grade cancer (ISUP 4 or 5). Twenty-five (22%) had known visceral metastases. Median baseline PSA was 197 ng per milliliter. All but four patients (97%) were pre-treated with docetaxel and 99 (80%) with ARTA. Baseline demographics are summarized in Table 1.

**Table 1:** Baseline patient characteristics

	n = 123
Age at KEES start, median (IQR), years	73 (68–77)
ECOG Performance status, n (%):	
0	13 (11%)
1	67 (55%)
2	38 (31%)
3	4 (3%)
Baseline PSA, median (IQR), ng/ml	197 (64–535)
Baseline ALP, median (IQR), mkat/l	2 (1–5)
Visceral metastases, n (%)	25 (22%)
Bone metastases, n (%)	112 (92%)
ISUP, n (%):	
1–3: Gleason < 8	49 (41%)
4–5: Gleason 8–10	71 (59%)
Curative treatment, n (%)	33 (27%)
Metastatic at diagnosis, n (%)	80 (66%)
Line of treatment*, n (%)	
2	28 (23%)
3	45 (37%)
≥ 4	50 (41%)
Prior docetaxel, n (%)	119 (97%)
Prior cabazitaxel, n (%)	35 (28%)
Prior ARTA, n (%)	99 (80%)
Prior radium-223, n (%)	14 (11%)

ECOG: Eastern cooperative oncology group, PSA: prostate-specific antigen, ALP: Alkaline phosphatase, ISUP: International society of urological pathology, ARTA: androgen-receptor targeting agents, IQR: interquartile range. *Excluding androgen-deprivation therapy.

### Treatment efficacy

At time for data cut-off, four patients were alive and one patient was still on treatment. Median time on KEES treatment for the whole cohort was 4.3 months, ranging from 3 days to 64 months, and median follow-up time 12.1 months. Thirty-one patients (25%) continued treatment at least one month beyond progression, for a median time of 3.4 months.

In the primary study population, median OS for patients pre-treated with at least one taxane and an ARTA was 12.3 months (95% CI 10.1–15.0 months) and median PFS was 4.4 months (95% CI 3.8–5.5 months). Biochemical response was achieved in 26 patients (29%), while any PSA reduction in 59 patients (65%).

In the overall study population, median OS was 12.1 months (95% CI 10.3–14.3 months) and median PFS was 4.3 months (95% CI 3.4-5.0 months). Biochemical response occurred in 38 patients (32%), and any PSA reduction in 75 patients (64%) (Table 2).

**Table 2:** Treatment efficacy

	Pretreated with a taxane and ARTA “Modern” population		Overall population	
OS, median (95% CI), months	12.3 (10.1-15-0)	95	12.1 (10.3–14.3)	123
6-month	82%		82%	
12-month	53%		50%	
24-month	17%		17%	
PFS, median (95% CI), months	4.4 (3.8–5.5)	94^×^	4.3 (3.4-5.0)	122^×^
6-month	37%		35%	
12-month	16%		16%	
24-month	5%		7%	
PSA response, n (%):		91^*^		117^*^
No	65 (71%)		79 (68%)	
Yes	26 (29%)		38 (32%)	
Any PSA reduction, n (%)		91^*^		117^*^
No	32 (35%)		42 (36%)	
Yes	59 (65%)		75 (64%)	

OS: overall survival, PFS: Progression-free survival, PSA: prostate-specific antigen, CI: confidence intervals, ARTA: androgen-receptor targeting agents. ^×^ One patient with missing data. *Four patients with missing data in the “modern” and six in the overall population.

**Fig. 1:**
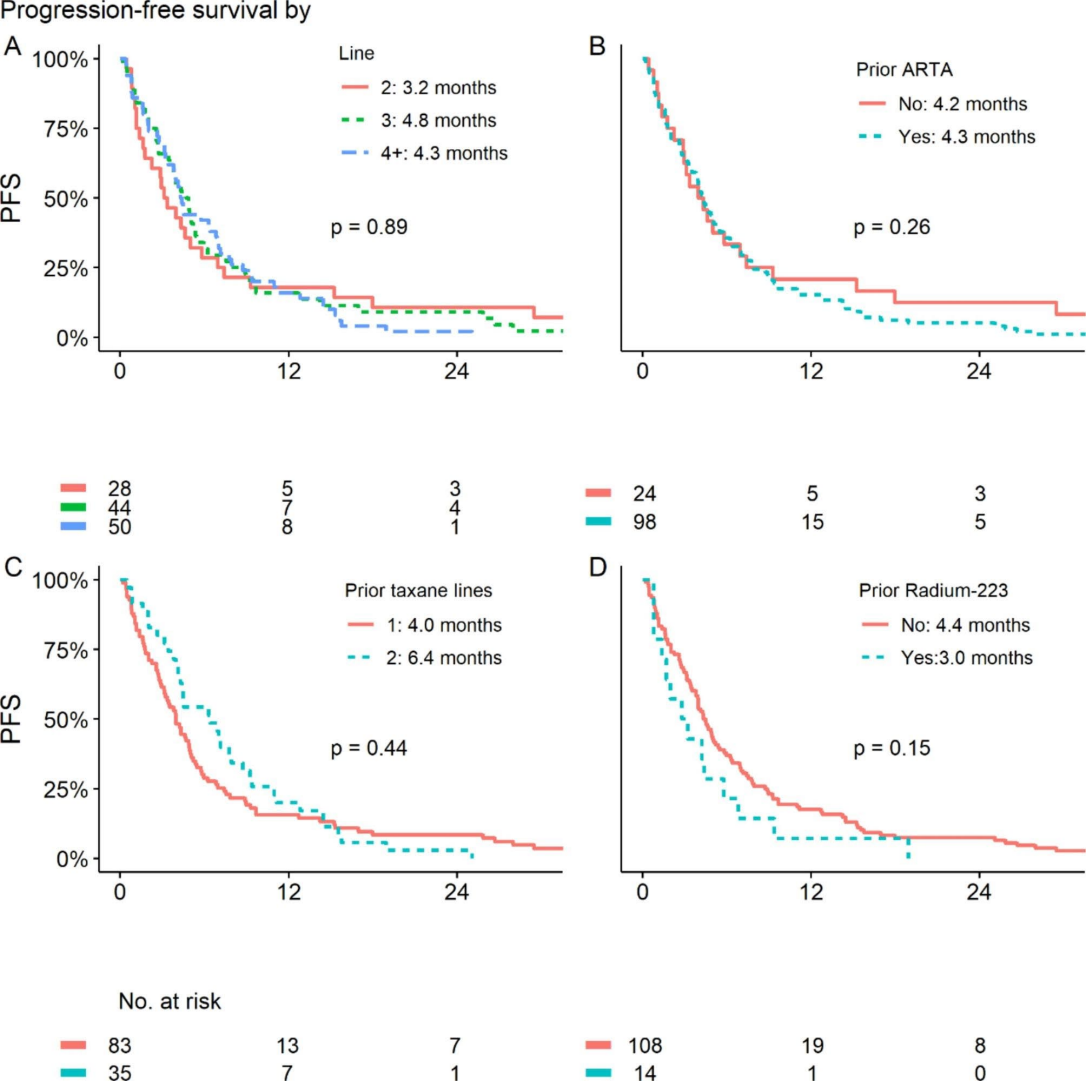
Progression-free survival (PFS) was independent of line (A) and of type of prior treatments (B-D). As only four patients were taxane-naïve, this group is not shown separately. ARTA: androgen-receptor targeting agents

**Fig. 2:**
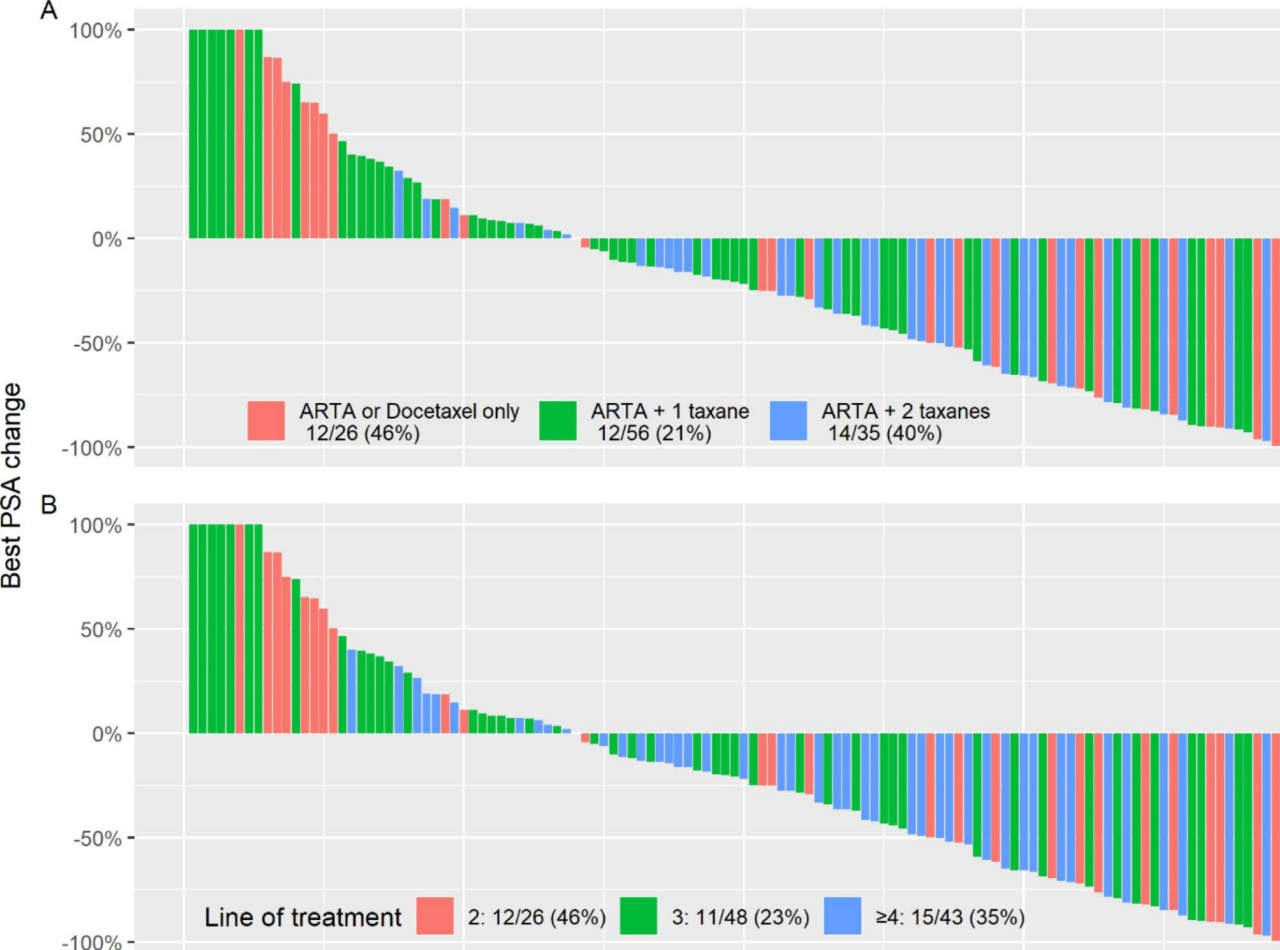
Best PSA change, truncated at 100%, by prior treatment type (A) and line of treatment (B). ARTA: Androgen-receptor targeting agents

PFS was largely similar regardless of treatment line (3.2 vs. 4.8 vs. 4.3 months for 2nd, 3rd and ≥ 4th line, respectively, p = 0.89), and of prior use of one or two taxanes (4.0 vs. 6.4 months, p = 0.44), ARTA (4.3 vs. 4.2 months, p = 0.26), or radium-223 (3.0 vs. 4.4 months, p = 0.15) (Fig. 1). Biochemical response was seen across all subgroups (Fig. 2).

In eight cases of patients with thrombotic events, estramustine was excluded from the initial combination, or stopped within the first treatment month. PFS did not differ for patients treated vs. not treated with estramustine (4.3 vs. 3.7 months, p = 0.53), however there was a trend towards more substantial median PSA reduction in the first group (-25% vs. -5%, p = 0.19). None of the patients treated without estramustine experienced a PSA reduction of > 50%, with the difference being borderline significant (35% vs. 0%, p = 0.052). Only one out of eight (13%) patients discontinued treatment for toxicity-related reasons and hematological toxicity appeared modest (one case of leukopenia grade 2, no cases of neutropenia and thrombocytopenia noted), however no formal comparison could be made due to the limited number of patient in this subgroup.

### Univariable and multivariable analysis

In a univariable analysis ECOG PS (reported as 0–1 versus ≥ 2, HR = 3.00, 95% CI 1.99–4.53, p < 0.001) and ALP as a continuous variable (HR = 1.06, 95% CI 1.02–1.11, p = 0.001), but not age, PSA, Gleason score, line of treatment, time from ADT to CRPC or treatment line, were significant prognostic factors for OS. All variables were entered in a multivariable analysis, stratified by year of treatment start (before vs. after 2015). An ECOG PS of ≥ 2 (HR = 2.63, 95% CI 1.61–4.28, p < 0.001) and increasing ALP (HR = 1.05, 95% CI 1.00-1.11, p = 0.040) remained unfavorable independent prognostic factors of OS (Table 3).

**Table 3:** Prognostic factors for overall survival

	HR (univariable)	HR (multivariable)
Age at KEES start, years	1.02 (0.99–1.05, p = 0.129)	1.02 (0.99–1.06, p = 0.130)
ECOG Performance status		
0–1		
≥ 2	**3.00 (1.99–4.53, p < 0.001)**	**2.63 (1.61–4.28, p < 0.001)**
Line of treatment		
2–3		
≥ 4	1.24 (0.85–1.81, p = 0.263)	1.44 (0.91–2.28, p = 0.123)
Baseline PSA, ng/ml	1.00 (1.00–1.00, p = 0.763)	1.00 (1.00–1.00, p = 0.350)
Baseline ALP, mkat/l	**1.06 (1.02–1.11, p = 0.001)**	**1.05 (1.00-1.11, p = 0.040)**
ISUP		
1–3: Gleason < 8		
4–5: Gleason 8–10	1.04 (0.71–1.51, p = 0.849)	1.18 (0.78–1.79, p = 0.437)
Visceral metastases		
No		
Yes	1.13 (0.72–1.80, p = 0.591)	0.89 (0.54–1.47, p = 0.660)
ADT to CRPC, years	0.99 (0.92–1.07, p = 0.832)	0.99 (0.91–1.09, p = 0.895)
Stratification: start before/after 2015		

ECOG: Eastern cooperative oncology group, PSA: prostate-specific antigen, ALP: Alkaline phosphatase, ISUP: International society of urological pathology, ADT: androgen deprivation therapy, CRPC: castration-resistant prostate cancer, HR: hazard ratio. Values in bold are significant.

### Treatment tolerability and toxicity

Thirty-eight patients (31%) discontinued treatment due to adverse events (AE) and/or worsened ECOG PS. Five of those interruptions occurred on treatment post progression. Main AEs included bone marrow toxicity, fatigue, infection, nausea and elevated liver function tests. Twenty patients (16%) developed thromboembolic events and 43 patients (35%) required hospitalization for any cause (including non-treatment related causes) up to one month after treatment discontinuation. Twenty-six patients (21%) had infection of any cause requiring antibiotics/antivirals (including urinary tract antiseptics) but only two cases of febrile neutropenia were noted. Most common hematologic toxicity was anemia, which was probably multifactorial in this heavily pre-treated population, whereas thrombocytopenia was the hematologic toxicity mostly related to treatment interruptions (nine cases of Grade 3 events). Four deaths were reported under treatment, one was in retrospect due to progression, and three infection-related; only one of the infection-related deaths occurred to a patient with grade 3 neutropenia. AEs are summarized in Table 4.

**Tabl﻿e 4:** Toxicity

	N = 122^×^
**Reasons for treatment discontinuation, n (%):**	
Death	4 (3%)^*^
Patient preference	2 (2%)
Plan	3 (2%)
Progression	75 (62%)
Toxicity or worsened performance status	38 (31%)
**Toxicities leading to discontinuation, n (%):**	
Bone marrow toxicity	5 (13%)
Cerebral bleeding	1 (3%)
Fatigue	7 (18%)
Infection	6 (16%)
Elevated liver function tests	6 (16%)
Nausea	6 (16%)
Other	7 (18%)^+^
**Leukopenia, max grade, n (%):**	
0	83 (68%)
1	13 (11%)
2	7 (6%)
3	16 (13%)
4	3 (2%)
**Neutropenia, max grade, n (%):**	
0	90 (76%)
1	7 (6%)
2	9 (8%)
3	9 (8%)
4	3 (3%)
**Thrombocytopenia, max grade, n (%):**	
0	84 (69%)
1	23 (19%)
2	5 (4%)
3	9 (7%)
**Anemia, max grade, n (%):**	
0	11 (9%)
1	54 (44%)
2	47 (39%)
3	10 (8%)
Febrile neutropenia, n (%)	2 (2%)
Infections under treatment, n (%)	26 (21%)
Thromboembolic events, n (%)	20 (16%)
Hospitalization, n (%)	43 (35%) ^**^

× One person with missing toxicity data *One case of herpes encephalitis 2 weeks after treatment start, with normal white blood cells/neutrophils, uncertain correlation. One patient died of S. aureus sepsis diagnosed three days after treatment start, unlikely correlated to treatment. One death secondary to infection/pulmonary embolism, with grade 3 neutropenia, likely treatment-related. One death on treatment, likely secondary to progression. ^+^Other: Diarrhea (3) Headache (1, likely secondary to meningioma), worsened renal function (1), stroke (2). ** Detailed data available for one of the participating centers: Infection (7), Nausea (2), pulmonary embolism (1), hematuria (2), pathologic fracture (1), worsened secondary to progression (1), and not specified (7).

## Discussion

The present study shows encouraging activity of KEES, a metronomic chemo-hormonal combination of cyclophosphamide, etoposide, estramustine, ketoconazole and low dose prednisolone in heavily pre-treated patients with advanced mCRPC. Treatment resulted in a PSA response rate of 29%, with a median OS of 12.3 months (95% CI 10.1–15.0 months) and a median PFS of 4.4 months (95% CI 3.8–5.5 months) in the primary analysis population of docetaxel and ARTA-pretreated patients, which is representative of the modern treatment landscape.

KEES was developed as a rescue treatment for mCRPC patients ineligible for other established treatment options in a small prospective cohort of 17 patients, who had not been previously treated with docetaxel or newer antiandrogens. In the study by Jellvert et al., 59% of patients achieved a PSA reduction of > 50%. Thrombocytopenia and anemia were the main toxicities in this early pilot. One study fatality was reported, although it was unclear whether this was treatment related or not [40].

Multiple small retrospective studies have evaluated the role of metronomic chemotherapy, mostly single cyclophosphamide or cyclophosphamide-based combinations, in mCRPC (Table 5). The bRR range from 5 to 82% in the group of patients not previously exposed to docetaxel or ARTA [27, 28, 30, 35, 36, 38–40]. In patients exposed to one of those two previous lines of treatment, the bRR is limited to 20–51% [20, 21, 23–26, 31–34, 37]; and only 15–16% in patients pre-treated with both treatment modalities (ARTA and docetaxel) [22, 29]. Median PFS ranges from 3 to 6 months in studies with pre-exposure to docetaxel or ARTA, with the exception of one study reporting a median PFS of eleven months; and 4-5.1 months in studies with pre-exposure to both classes of drugs. Median OS ranges from 11 to 28 months for patients treated with only one agent and is identical at 8.1 months in the two studies with populations pre-treated with both agents.

**Table 5:** Studies with metronomic chemotherapy

Trial	Treatment	n	Prior docetaxel	Prior ARTA	PFS (mo)	OS (mo)	bRR
**Prior docetaxel/ARTA in < 50%**							
Glode, 2003	CTX + dexamethasone	34	0%	0%	9*	NR	69%
Lord, 2007^+^	CTX	80	0%	0%	7,5	NR	5%
Yashi, 2014	CTX + dexamethasone	24	46%	4%	5	19	33%
Fontana, 2010	CTX + celecoxib + dexamethasone	29	41%	0%	NR	NR	45%
Jeong, 2017	CTX + celecoxib + dexamethasone	49	45%	0%	3,9	13,3	39%
Derosa, 2014	Docetaxel + prednisone + CTX + celecoxib^±^	41	0%	0%	14,9**	33,3	82%
Jellvert, 2011	KEES	17	0%	0%	NR	NR	59%
Tralongo, 2016	Vinorelbine	14	0%	0%	8,6***	NR	NR
**Prior docetaxel**							
Nelius, 2009	CTX + dexamethasone	17	100%	0%	NR	24	24%
Ladoire, 2010	CTX + prednisolone	23	100%	0%	6	11	26%
Dickinson, 2012	CTX + dexamethasone	28	50%	0%	4	NR	NR
Barroso-Sousa, 2014	CTX + prednisone	40	100%	0%	3	11,9	20%
Calvani, 2019	CTX + dexamethasone/prednisone	37	62%	13%	11	28	51%
Fontana, 2009	CTX IV + oral + celecoxib + dexamethasone	28	68%	0%	3**	21	32%
Kubota, 2017	UFT, cisplatin, dexamethasone^±^	25	100%	0%	6****	14	20%
Wang, 2015^++^	Lenalidomide + CTX	25	100%	0%	NR	20	32%
Gebbia, 2011	CTX + methotrexate	60	100%	0%	5,2	11,5	25%
Di Desidero, 2016	Vinorelbine + dexamethasone	41	85%	0%	4	17,5	35%
**Prior ARTA**							
Dabkara, 2018	CTX + prednisolone	18	33%	61%	4,7	NR	44%
**Prior Docetaxel + ARTA**							
Knipper, 2019	CTX + prednisolone	14	71%	100%	5,1	8,1	15%
Caffo, 2019	CTX	74	100%	100%	4	8,1	16%
Present study	KEES	123	97%	80%	4,3	12,1	32%
Present study, pretreated	KEES	95	100%	100%	4,4	12,3	29%

CTX: cyclophosphamide, UFT: Tegaful-uracil, ARTA: Androgen receptor targeting agents, NR : Not reported, PFS: progression-free survival, OS: overall survival, mo: months, bRR: biochemical response rate. Eligible if at least^+^two cycles ^++^one cycle was given^±^Combinations with classical chemotherapy *+50% from nadir if > 50% reduction, + 25% otherwise **+50% from nadir and at least 5 ng/ml ***+50% from baseline **** Biochemical progression: 50% increase.

Our study included 123 patients from a heavily pre-treated population; ninety-five of those had received treatment with at least a taxane and an ARTA. Our PFS of 4.4 months was in line with the two studies including patients treated with both a taxane and an ARTA, whereas our biochemical response rate of 29% and OS of 12.3 months were considerably higher. Interestingly, some patients achieved sustained responses, with 16% having no evidence of disease progression at one year.

KEES appears effective in multiple lines of treatment, regardless of previous exposure to taxanes or ARTA, in contrast to the use of single metronomic cyclophosphamide, which seems more beneficial early in the disease trajectory. In a study by Calvani et al., median PFS in subjects treated with metronomic single cyclophosphamide was almost doubled for docetaxel-naïve patients compared to docetaxel-pre-treated ones (19 vs. 11 months) [20]. Jeong et al. reported comparable time to PSA progression for the combination of cyclophosphamide and celecoxib before versus after docetaxel (5.5 vs. 4.9 months), whereas the difference in OS was more pronounced (15.0 vs. 9.7 months) [38]. A combination that is effective in late treatment lines might prove relevant in the modern treatment landscape, where most patients have been treated with at least one type of taxane and one ARTA.

In our experience, nausea and mild gastrointestinal discomfort might occur during the second week of treatment. Nausea was an issue especially during the initial study period, as antiemetics were not routinely prescribed. Thromboembolic events, a major concern during treatment with estramustine [44], occurred in 16% of the patients. Of note, peripherally inserted central catheters, which have been associated with up to eight times higher risk of catheter-related thrombosis [45] were commonly used during the study period; prophylactic anticoagulation treatment was not offered. A third of all patients discontinued treatment because of side effects and/or worsened performance status. In retrospect, the high discontinuation rate may be partly caused by patient selection, as KEES was often prescribed to patients ineligible for other treatments. All four patients with a baseline ECOG PS of three stopped treatment within the first month. In addition, two-thirds of the patients had grade 2 or worse anemia and three patients had grade 3 thrombocytopenia at treatment start, which most likely would have excluded them from treatment with conventional chemotherapy.

A major limitation of our study is that the relative impact of all components of the KEES regimen remains unknown. The regimen was developed as an orally administered modification of an older chemo-hormonal combination. In a contemporary study population, pretreated with at least a taxane and an ARTA, the bRR of 29% compared favorably with the two studies using metronomic cyclophosphamide (which had bRR of 15–16%), implying that the combination might be more active than single cyclophosphamide. In a small subgroup of patients not receiving estramustine, we could see a trend towards worse bRR, suggesting that estramustine might contribute to the regimen’s overall activity. However the small sample size precludes any firm conclusions. It must also be noted that the availability of oral ketoconazole might be limited in some countries. In this study, no patients were treated without ketoconazole, and we could not evaluate the relative importance of this drug for the efficacy of the combination.

The study has several limitations related to its retrospective nature. The treatment paradigm changed during the study period, with the addition of cabazitaxel, ARTA and radium-223, and KEES was switched from a second line treatment to the fourth or fifth line. In order to study a more homogeneous scenario, comparable to a typical “modern” population, primary analysis was therefore limited to patients pre-treated with both a taxane and an ARTA. Efficacy endpoints were not worse in this group. Recently, Lutetium-PSMA has been approved, with bRR of 46–66% and a four-month survival benefit compared to standard care [11, 46]. The role of any metronomic treatment without survival benefit proven in a randomized trial, including KEES, is limited to patients where treatments with proven survival benefit have been exhausted, or are not eligible. Imaging was not always performed at regular intervals during the study initiation. As information on AEs was collected retrospectively by review of medical records, severity of nausea/abdominal discomfort and fatigue could not be reliably reported. However this is to our knowledge the largest study to date on patients with metronomic chemotherapy of any kind in prostate cancer, and one of only three reflecting the current treatment landscape. The key strength is the true real-world approach, meaning that all patients regardless of socio-economic status, age, or comorbidity were included. The Swedish health care system is publicly funded and in the present area, the two centers included in this study are the only facilities offering cancer chemotherapy. This means that patient selection bias is highly unlikely to have influenced the results.

## Conclusions

In conclusion, we suggest that the metronomic chemo-hormonal combination (KEES) is a reasonable option in patients with heavily pre-treated mCRPC, especially in those with good ECOG PS and lower baseline ALP. Toxicity was manageable and bRR was higher compared to published data of other late-line metronomic treatments in a similar mCRPC population. Future prospective trials are needed to further explore and confirm the value of KEES in mCRPC.

## Data Availability

Additional data are available at reasonable request to the corresponding author.
